# Configurable 2D nano-flows in mesoporous films using paper patches†

**DOI:** 10.1039/c7ra13691a

**Published:** 2018-02-08

**Authors:** M. Mercuri, R. Gimenez, C. L. A. Berli, M. G. Bellino

**Affiliations:** Comisión Nacional de Energía Atómica, CONICET, Departamento de Micro y Nanotecnología Av. Gral. Paz 1499 1650 Argentina mbellino@cnea.gov.ar; INTEC (Universidad Nacional del Litoral-CONICET) Predio CCT CONICET Santa Fe RN 168 3000 Santa Fe Argentina cberli@santafe-conicet.gov.ar

## Abstract

Designing and controlling spontaneous imbibition is becoming a key requirement for advanced devices, presenting a substantial scientific and engineering challenge. Here we describe an approach that allows directional imbibition into designed geometries. A set of custom domains based on paper microfluidics mold nano-imbibition in user-defined shapes such as curvatures, corners, and vertices into mesoporous thin films; enabling localized chemical reactions with programmable designs. The method also achieves nano-size filtration, allows the generation and delivery of reagent gradients in a nanofluidic fashion, and it can be used as a reactor for the synthesis of patterned metallic nanoparticle arrays. By using this easy-to-build hybrid platform, users can create functional nanofluidic domains in custom geometries and perform spatially shaped chemistry. The ability to integrate mesoporous nanofluidic generation and paper-based microfluidics has made the hybrid system an exciting candidate for versatile nanoflow applications.

## Abstract 2

Mesoporous film-based nanofluidics has been converted into a versatile technique by using cut paper.

## Graphical abstract

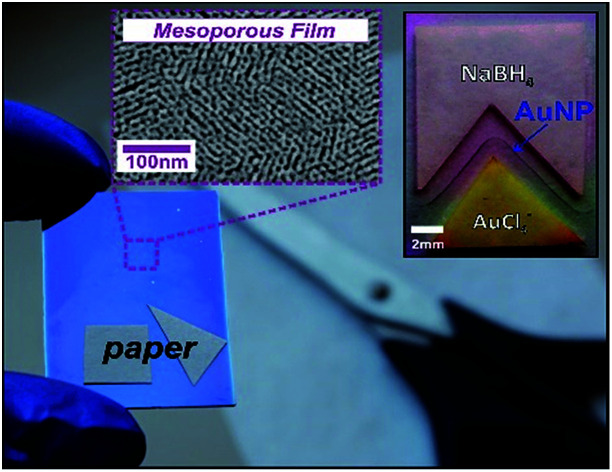

## Introduction

Spontaneous imbibition enables the propelling of flows because of the preponderance of capillarity at small length scales.^1^ One challenge in flow dominance is the design of imbibition to attain total control of the arrangement of fluids. Such fluidic transport attracts interest from the fields of fundamental research, medicine, and biotechnology. Currently there is increasing activity in developing microfluidic paper-based devices,^2,3^ where passive pumping represents a practical advantage added to its extraordinarily low cost and compatibility with most (bio)chemical reactions. Paper-based microfluidics provides access to functional modules with overall or repeat unit dimensions ranging from tens of microns to centimeters.^4^ On the other hand, mesoporous films exhibit arrested fluid imbibition due to the balance between capillary filling and surface evaporation to the environment.^5^ This effect allows one the implementation of mesoporous film-based nanofluidics, which offers the possibility to manipulate ultrasmall amounts of liquid,^6^ and also serves as hubs for forming high-localized reactions.^7^ An additional advantage of this strategy is that the usually costly steps of sealing and tubing can be obviated in the fabrication of nanodevices. With the idea in mind to generate addressable nano-imbibition with increasing patterns of complexity and controlled reagents delivery, we have used mesoporous film based functionalities into designed paper-based scaffolds. In this study, we have achieved reliable design of the two dimensional imbibition of mesoporous films with programmed control over their geometry. The capabilities of this method are demonstrated by implementing chemical reactions in shaped fluidic domains, including nano-optical structure synthesis; attaining hierarchical selectivity in micro/nano filtration; and generating chemical gradients and subsequently unloading reagents to achieve preprogrammed reactions. Therefore, these on-demand imbibitions behave as smart nano-flows which provide a well-founded starting point for the exploration of more sophisticated chemical and biomedical applications.

## Results and discussion

### Shaped nanofluidic domains

**Fig. 1:**
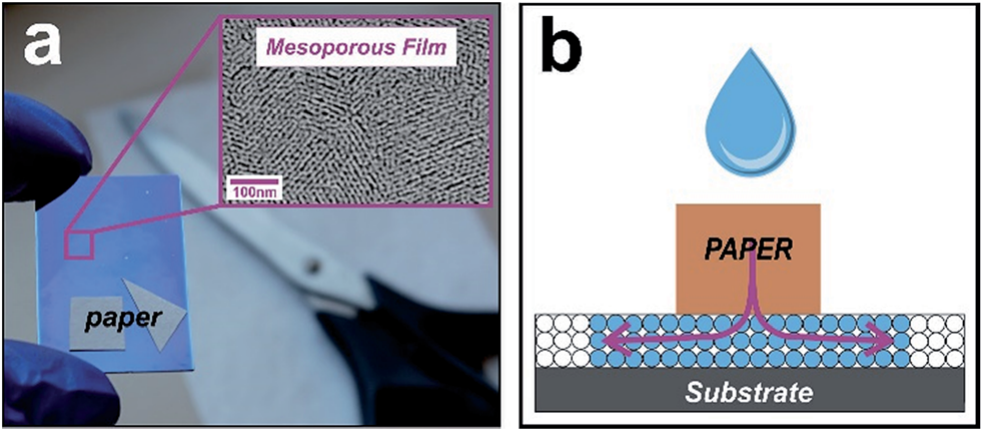
Concept for constructing nano-imbibition shapes (a) picture showing the hybrid paper-mesoporous system. The inset on the right reveals a scanning electron micrograph (SEM) of the mesoporous film composed of an array of nanopores. (b) Schematic illustration of the hybrid-system concept.

Controlling the spatial arrangement of spontaneous infiltration in nanoporous systems is a central goal for versatile devices. We then reimagined the open pit design principle^7^ from paper-based microfluidics. Instead of using sessile drops as reservoirs, now our approach uses imbibed papers. In this method, a paper with arbitrary shape assumes the role of a reservoir that is cut into a user-defined arrangement (sketched in Fig. 1). We explored the possibility of creating custom curvatures, corners, and vertices, in order to copy these forms into the fluid front of the liquid that infiltrates the underlying material. Our devices are based on a mesoporous oxide film grown on silicon or glass substrates using F-127 as a surfactant template, by combining sol–gel chemistry and evaporation-induced self-assembly.^5,8^ Fig. S1 in ESI† shows typical SEM and TEM images of the mesoporous silica and titania films used in this work, where the nanopores can be clearly observed.

**Fig. 2:**
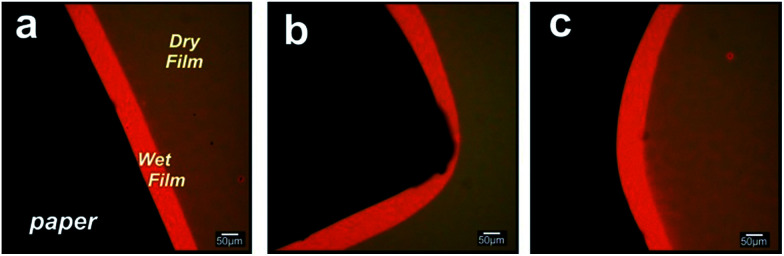
Microscopy study of the nanofluidic front design. Images show the imbibition front that replicate characteristic shapes from the cut paper in mesoporous titania films. (a) a 180° front, (b) a triangle and (c) an open concavity.

We then placed a series of paper shapes, a 180° straight front, an open concavity and a triangle on the surface of mesoporous films. When a drop is deposited on the paper the liquid from the paper reservoir enters the porous substrate and builds a halo at its periphery. This halo nucleates and grows *via* capillarity at their free boundaries which infiltrate into the nanoporous film. Imaging on each preformed paper corroborated the successful imbibition with the designed shape (see Fig. 2). The wetted region can be clearly seen because it produces a refractive index contrast in relation to the dry zone.^5^ The flat paper limits were more reproduced than the corners, as expected because of the paper adhesion constraint in the vertices. Together, the different shapes in Fig. 2 constitute a set of design motifs that could be combined to create larger, more complex patterns. It is worth mentioning that the hybrid system is modular and scalable, and can therefore be extended from the micrometers to centimeter scale according to the surface/area of the cut paper.

### Size-exclusion capabilities

**Fig. 3:**
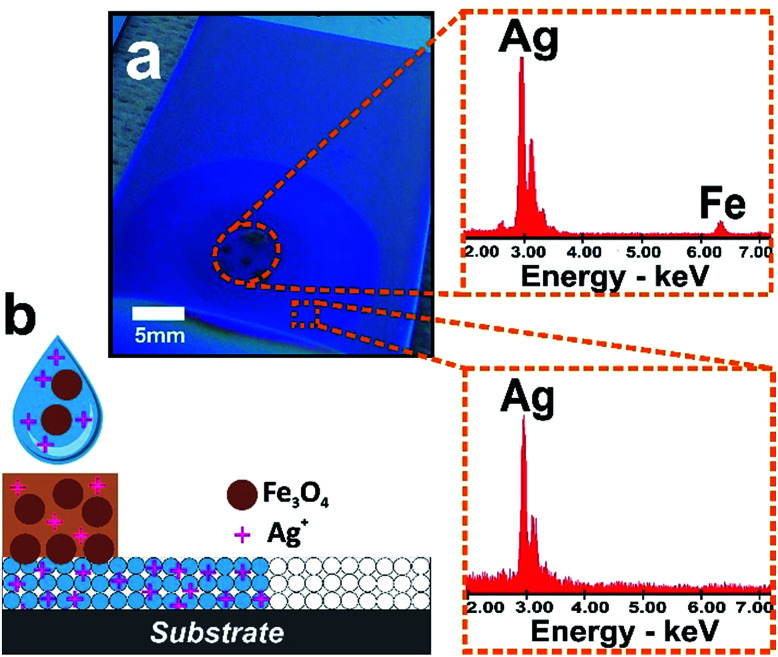
Mesoporous infiltration as selective functional membrane (a) image of mesoporous silica film after filtration process. EDS microanalysis showing the different chemical entities present in the capillary infiltrated mesoporous film regions (halo) and paper zone. Dashed circle corresponds to the zone where the paper was placed. (b) Schematic depiction of the size selective filtration processes taking place in the hybrid assembly.

In order to take advantage of the size-exclusion properties of the nanopores of our hybrid fluidic platform, we incubated a 5 mm in diameter paper-reservoir deposited on a silica mesoporous film with a mixture comprising an AgNO_3_ solution and iron oxide nanoparticles (nanocrystalline magnetite, Fe_3_O_4_ with an average size of 10 nm; leading to a narrow nanocluster size distribution in aqueous solution of *ca.* 35 nm. See ESI† for more details).^9^ After mesoporous infiltration the paper was removed and Energy Dispersive X-ray Spectroscopy (EDS) was used to quantitatively measure the presence of Ag and Fe in the different zones. The EDS data obtained from the paper and mesoporous zones revealed the presence of both Fe and Ag along the paper region and only Ag ions in the infiltrated mesoporous outside the paper zone (see Fig. 3). It is evident that the paper released all two species while the mesoporous film avoids the transport of the magnetite nanoparticles and enables the diffusion of Ag ions into the film, indicating that the system can filter as designed. The relatively large Fe_3_O_4_ nanoclusters are excluded from the small pore silica which however can act as a host layer for smaller entities such as Ag ions. This example illustrates that the synergic properties of paper-based microfluidics integrated with a mesoporous film solve the challenge of simultaneously achieving size selectivity and tuned nanoflow. With the wealth of mesoporous structures developed is possible to design the selectivity of these hybrid systems by varying the pore dimensions in the mesoporous film.

### Molded chemistry

**Fig. 4:**
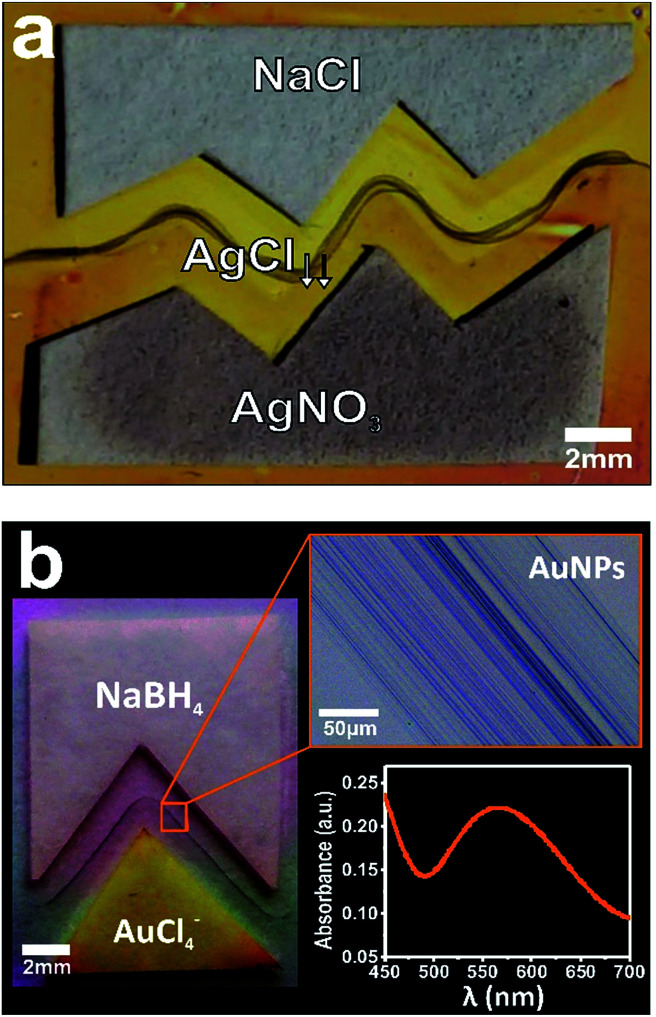
Molded chemistry assisted by hybrid paper-mesoporous system. (a) Picture of two papers reservoirs imbibed of NaCl (top) and AgNO_3_ (down) solutions in a mesoporous titania film on a silicon substrate exhibiting the molded AgCl line arising from paper cut. (b) Picture of two papers reservoirs imbibed of HAuCl_4_ (down) and NaBH_4_ (top) solutions in a mesoporous titania film on a glass substrate presenting the molded precipitation of Au nanoparticles. Insets: microscopy image of the localized Au nano-precipitation (upper frame) and ultraviolet-visible spectrum collected from gold nanoparticles (lower frame).

We further capitalized on this new hybrid system by expanding their capabilities to form localized reactions with programmable geometries. We then tested the possibility to localize a chemical reaction with user-desired shapes starting from custom shaped cut papers with a separating gap between them. To this end, we used the well-known Ag^+^ + Cl^−^ → AgCl reaction by depositing microliter drops of 1 M sodium chloride (NaCl) and 0.1 M silver nitrate (AgNO_3_) on both paper strips, respectively.^7^Fig. 4 shows the proper localization of silver chloride (AgCl) precipitation through the nanocapillary communication of the different paper-reservoirs, and more importantly demonstrating the capability of the hybrid method for molding chemical reactions at the nanofluidic connection. The large distances over which these shaped reactions extend demonstrate the overall robustness of this hybrid method. Although the method was designed to generate pointed structures formed by chemical reaction at the time of contact, we expected to (and finally did) observe an extended rounded precipitation shape because of the front roughening effect that takes place during spontaneous imbibition in porous media.^7^ Taking advantage of the latent properties described above for “drawing” with metallic nanoparticles, we used the process for transferring geometrical contours on a cut paper into gold nanoparticle arrays. Optical images in Fig. 4b display the creation of nanostructured gold patterns with arbitrary shape *via* salt reduction by depositing microliter drops of 0.1 M tetrachloroauric (HAuCl_4_) at pH = 4, and 1 M sodium borohydride (NaBH_4_) in each paper reservoir, respectively. The characteristic ultraviolet-visible spectrum (surface plasmon resonance) evidences that the precipitate is an extended nanoassembly of Au nanoparticles (see inset in Fig. 4b).^10^

Further, these reaction structures also form well-defined substructures that run parallel each other. This capability will allow for applications that require a precise spatial location of metallic nanospecies such as in advanced catalysis, nanoelectronic circuits, chemical and biological sensor devices, optical switches, *etc.*^11^ Additional investigation of the substructuration mechanism will prove useful in the development of this method for patterning complex opto-electronic nanostructures.

### Nanofluidic gradient-actuated reactions

**Fig. 5:**
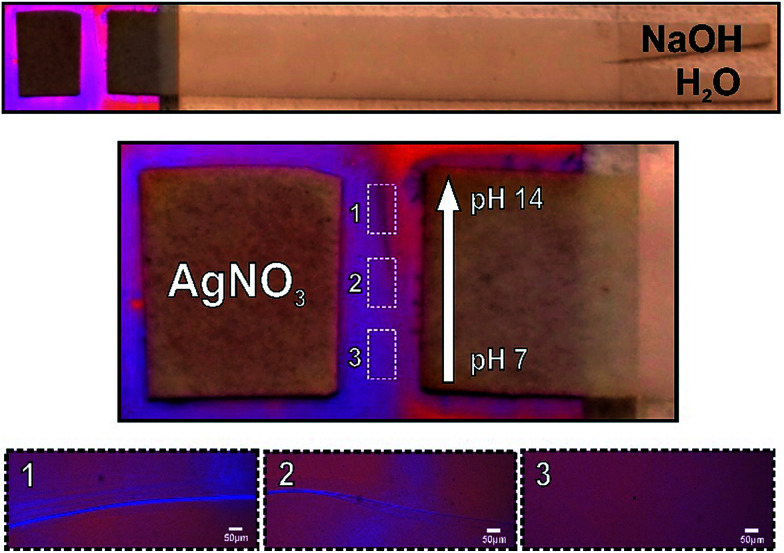
Nanofluidic gradient-actuated reactions. Photographs of a classical paper based gradient generator imbibed of water and NaOH solutions faced with a paper imbibed of AgNO_3_ solution on a mesoporous titania film exhibiting the localized precipitation in concordance with pH gradient. The inset optical microscopy images show blow-ups in the different pH zones.

Finally, in order to test the utility of the hybrid method for controlling the concentration of solutes across the mesoporous membrane, we conducted a pH gradient experiment by introducing a paper-based gradient generator. The simple format of a uniform strip with Y-shaped inlet ports was employed.^12^ Besides, here the system was laminated to avoid evaporation, leaving both ends (inlet ports and gradient exit) open to the atmosphere. Pure water and NaOH 1 M solution are added to each one of the inlet arms. The capillary-driven co-flow of solutions along the strip enables the dispersion of OH ions from the concentrated stream to the other, leading to a linear gradient that ranges from 7 to 14 at the strip end, perpendicular to the flow direction. This strip end was in contact with the mesoporous film, as shown in Fig. 5 (left panel). The use of such basic solutions to precipitate oxides is a long-standing practice in the field of material science.^13^ It should be mentioned that oxide precipitation in metal salt solutions is produced in high pH conditions. Here, we adapted this method to test a localized gradient in pH into mesoporous film. We then performed the well-known 2 AgNO_3_(aq) + 2 NaOH(aq) → Ag_2_O(s) + 2 NaNO_3_(aq) + H_2_O(l) reaction and analyzed the response of the precipitation signal to formation of the pH gradient by the paper system. In fact, this precipitation was actuated by imposing a pH gradient along the nanofluidic contact interface. As expected, this assay produced a precipitation reaction within the interface only in the high pH areas (see Fig. 5). The analysis of optical microscopy images makes evident that precipitation depends on the concentrations of OH^−^ in the nanopores, which has been regulated by using the paper-based pH gradient. These observations confirmed the proper graduation of OH^−^ through the nanopores, thus demonstrating the utility of the hybrid method for producing space-selective solute concentration into the infiltrated fluid. Paper-based generation of concentration gradients could be used to direct the movement of cells^14^ on the mesoporous substrates^15^ or used for drug delivery by nanodispensers for dose testing.

## Conclusion

We successfully developed a versatile multiscale fluid-handling strategy based on nanopores-paper-scissors. Together the results here presented demonstrate that a hybrid platform of paper/mesoporous film is able to mold fluid transport and chemical reaction shapes with synergistic features such as size selectivity and the capability of creating gradients, even if the procedure has not been yet fully optimized. Although several types of nano-hybrid paper devices have been described, the most significant difference with our proposal is that they are mostly obtained by modification of paper substrate, which demonstrates the high simplicity of the strategy here presented. We created a diversity of fluid-fronts in the spontaneous mesoporous film imbibition by designing custom paper shapes only using a cutter, and by means of these hybrid structures, we designed nano-size filtration and user-defined shapes for chemical reactions. Our approach for creating paper/mesoporous film hybrid devices affords attractive capabilities with respect to positioning target features in custom nanostructured frameworks, and offers the additional advantage that all structural components are inexpensive.

Apart from the possibility for scalable mass production and the straightforward of exploiting nanopore functionalization^16–19^ and confinement,^20,21^ our method could therefore enable novel applications that so far have not been amenable to current methods in paper-based microfluidic. This heterogeneously integrated approach provide indeed synergetic functionalities to systems that could be important in various fields of application, including not only those suggested by the systems reported here but also others such as devices with integrated electronics,^22^ chemical and biological sensor systems that incorporate unusual nanomaterials with conventional paper based microfluidics, and photonic and optofluidic porous silicon structures.^23^ Furthermore, the compatibility of this approach with nano-optical detection from synthesis of metallic nanoparticles in arbitrary patterns may create additional opportunities for devices that have unusual form factors as key features.^24^

## Experimental

### Mesoporous thin films synthesis and characterization

Crack-free mesoporous titania and silica films were synthetized using sol–gel technology and evaporation-induced self-assembly technique. For this purpose, Pluronic F127 was used as the polymeric template. Titania films were dip coated at 3 mm s^−1^ on silicon and glass substrates at 20% relative humidity, while silica films were deposited on silicon at 1.5 mm s^−1^ withdrawal rate. Precursor solution for titania films was composed of TiCl_4_ : EtOH : H_2_O : F127 with a molar ratio of 1 : 40 : 5 : 0.005 and the initial solution for silica films was the mixture TEOS : EtOH : H_2_O : F127 with a molar ratio of 1 : 40 : 5 : 0.04. After deposition, samples were calcined in air at 450 °C for 10 min by a fast-firing process in order to remove the template. Samples were characterized with a Zeiss Leo 982 Gemini Scanning Electron Microscope (SEM) coupled to an X-ray microanalyzer and using a Phillips CM 200 Transmission Electron Microscope (TEM).

### Hybrid paper-mesoporous film system

Hybrid system was built with mesoporous titania and/or silica films and Whatman No. 1 filter paper used as received. Paper strips with different geometries were shaped using a drawing cutter and directly deposited on the surface of the mesoporous film. Molded chemical reactions for AgCl precipitation were performed using paper reservoirs imbibed of 20 μl of silver nitrate (AgNO_3_) 0.1 M and 20 μl of sodium chloride (NaCl) 1 M, spaced 5 mm apart. In case of Au nanoparticles precipitation, 10 μl of tetrachloroauric (HAuCl_4_) 0.1 M at pH 4 and 20 μl of sodium borohydride (NaBH_4_) 1 M were used. UV-visible spectrum was obtained using a UV-1800 Shimadzu Spectrophotometer. Precipitation zone in microscope areas and the studies of the imbibition fronts were followed by a Mitutoyo FS70 microscope. Images were recorded using a high-resolution digital camera. Nanofluidic gradient-actuated reactions were carried on using a uniform strip with Y-shaped inlet ports. In this case, the paper was laminated to avoid evaporation, leaving both inlets and the gradient outlet open to atmosphere. To generate the pH gradient on the paper strip: 30 μl of NaOH 1 M and 30 μl of deionized water were added to each inlet arms. Then the system was brought in contact to the mesoporous film and faced to a paper reservoir containing 10 μl of AgNO_3_ 0.1 M at pH 9. Gradient precipitation reaction was observed under the Mitutoyo FS70 microscope.

## Conflicts of interest

There are no conflicts to declare.

## Supplementary Material

RA-008-C7RA13691A-s001
