# Supplementary material for: Configurable 2D nano-flows in mesoporous films using paper patches
Source: RSC Adv. 2018 Feb 8;8(12):6414–8. doi: 10.1039/c7ra13691a (PMC9078350; doi:10.1039/c7ra13691a)
Supplement: RA-008-C7RA13691A-s001 [file RA-008-C7RA13691A-s001.pdf]

Electronic Supplementary Material (ESI) for RSC Advances.
This journal is © The Royal Society of Chemistry 2018

Supplementary Information

Configurable 2D Nano-Flows in Mesoporous Films Using Paper Patches

Magalí Mercuri,[1] Rocio Gimenez,[1] Claudio L. A. Berli,[2,] and Martín G. Bellino[1,*]*

1- Comisión Nacional de Energía Atómica, CONICET, Av. Gral. Paz 1499, San Martín, Buenos Aires, Argentina.

2- INTEC (Universidad Nacional del Litoral-CONICET) Predio CCT CONICET Santa Fe, RN 168, 3000 Santa Fe, Argentina.

Characterization of titania and silica mesoporous films

Titania and silica films were characterized by Scanning Electron Microscopy (SEM) and Transmission Electron Microscopy (TEM):

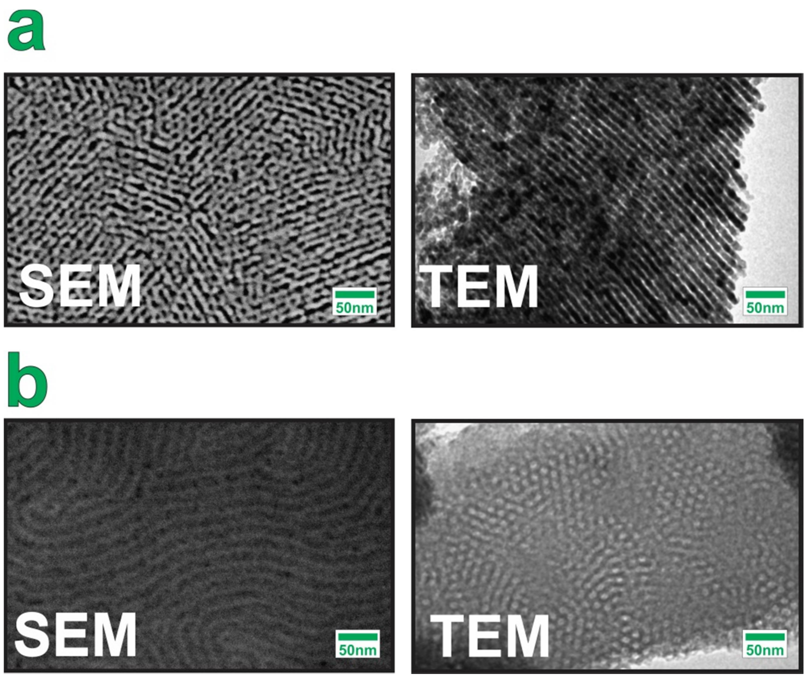

Figure S1: Mesoporous Thin Films characterization. SEM and TEM micrographs of titania (a) and silica (b) thin films.

Synthesis and characterization of Magnetite Nanoparticles:

Magnetite nanoparticles were synthetized by co-precipitation of a mixed solution of ferrous chloride ($FeCl_2.4H_2O$, 0.2M in HCl) and ferric chloride ($FeCl_3.6H_2O$, 0.1M in H_2O) through dropwise addition of NaOH 0.15M. Synthesis was made under N_2 atmosphere with vigorous agitation at room temperature; N_2 was blown into all solution during 15 minutes prior to reaction. In order to remove the residual ions, the obtained precipitate was centrifuged and washed five times with deionized water. Crystalline phases and the average crystallite size were obtained by X-Ray diffraction (XRD) using a Philips PW 3710 diffractometer with CuKa radiation and a graphite monochromator. Approximate crystallite size (10±1 nm) was determined with Scherer equation. The average size of magnetite nanoparticles observed by SEM was in good agreement with the XRD measurements. Size distribution of nanoparticle clusters (average size = 35nm) was determined using Dynamic Light Scattering (DLS).

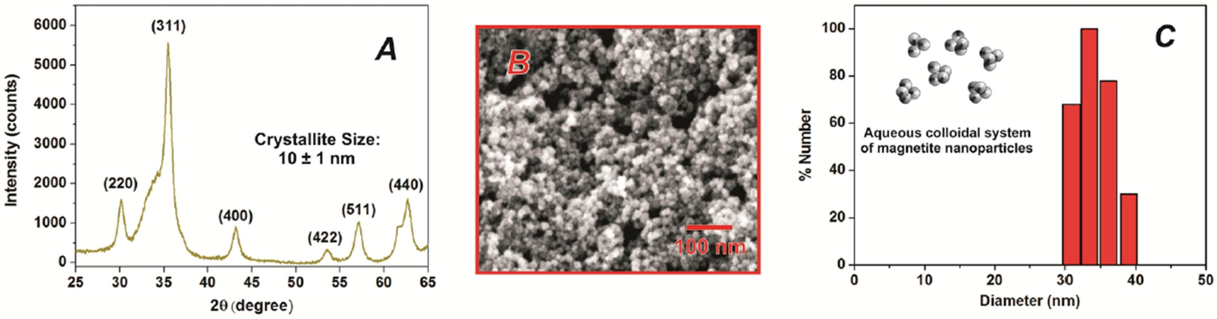

Figure S2: Magnetite Nanoparticle characterization. A) X-ray diffraction (XRD) spectrum, B) SEM micrograph of the nanoparticles and C) DLS measurement of colloidal dispersion showing the clusters size distribution.
